# Examining factors contributing to the socioeconomic inequalities in handgrip strength among older adults in India: a decomposition analysis

**DOI:** 10.1038/s41598-023-50613-3

**Published:** 2024-01-03

**Authors:** Manacy Pai, T. Muhammad

**Affiliations:** 1 Department of Sociology and Criminology, Kent State University, Kent, OH 44242 USA; 2 Pennsylvania State University, University Park, PA 16802 USA

**Keywords:** Geriatrics, Biomarkers, Health care

## Abstract

The purpose of this study was to examine (a) the socioeconomic status (SES)-related inequalities associated with handgrip strength (HGS); and (b) the extent to which several demographic, health, and behavioral factors contributed to such SES disparities in HGS among older adults in India. Data were drawn from the 2017–2018 wave 1 of the Longitudinal Ageing Study of India (LASI). The study sample included 27,707 older adults (13,199 men and 14,508 women) aged 60 years and older. HGS was assessed using a handheld Smedley's Hand Dynamometer with a cut-off of 19.5 kg for men and 12.5 kg for women. Bivariate analysis showed the weighted percentage distribution of weak HGS across respondent characteristics. Multivariate logistic regression assessed factors linked to weak HGS. The concentration curve and index (CCI) was used to determine the inequalities in the prevalence of weak HGS by wealth index scores. Wagstaff's decomposition approach was used to test the contribution of each explanatory variable to weak HGS. Around 9% of older adults in this study reported a weak HGS. It was significantly higher among those aged 80 or older (19.21%) and males (15.55%). Weak HGS was concentrated among older adults from poor SES (CCI: 0.05, p < 0.001). A higher percentage of wealth-based inequality in weak HGS was explained by being underweight (38.83%), belonging to the richest wealth quintile (27.95%), and having a higher subjective social status (32.20%). Moreover, about 23.29% of the inequality in weak HGS was explained by Western region and 22.54% by female gender. Additionally, having a secondary level of education explained a higher percentage (22.09%) of inequality, followed by current working status (− 20.68%). Rural residence (13.08%), limitations in instrumental activities of daily living (IADL) (12.21%), and engagement in yoga-related activities (11.55%) explained a higher percentage of wealth-based inequalities. The findings provide evidence of significant SES-related inequalities in HGS and the contribution of various demographic, health, and behavioral factors to such inequality. As such, public health policies and programs focusing on reducing the burden of disability must consider the contribution of social and economic equity to the preservation of muscle strength among older adults.

## Introduction

Decline in muscle strength, though an anticipated challenge associated with normal aging^1,2^, is a public health concern given that it is associated with greater dependence in activities of daily living, and an elevated risk of functional disability, which may mean decreased autonomy and quality of life and increased likelihood of aging “out of place”^3–6^. Moreover, weak muscle strength is predictive of greater likelihood of disease complications, protracted post-surgery recovery time, longer hospital stays, increased mental distress, and even cognitive decline and premature mortality^2,7–10^.

Handgrip strength (HGS) is the most commonly used way to evaluate muscle strength among older adults^1,9^. It captures age-based fluctuations and fluctuations in biological function and, consequently, is a marker of biological vitality and an indicator of muscle potency^3^. HGS is an easy-to-use, reliable clinical test to assess general physical functionality^11^, particularly in developing nations like India, because of its predictive value, non-invasive nature, consistency and simplicity in measurement, portability, and affordability.

Like other morbidities, HGS is related to various indicators of socioeconomic status (SES), including education, employment, income, and wealth^12–16^. For instance, in their research on aging adults in 11 European nations, Mohd Hairi et al. observed that while education, occupation, income, and wealth are all linked with HGS in men, education and wealth are more consequential for HGS in women^14^. In their study on older Indonesians, Pengpid and Peltzer observed a statistically significant positive link between education and HGS among men^15^. Likewise, the Arokiasamy et al. found a significant and positive association between education and wealth and HGS among older adults in India^12^; and, in another study among older Koreans, researchers found that those who transitioned from working to non-working status reported an increased risk of weak HGS^17^.

In addition to SES, there are demographic, social, and behavioral factors found to influence HGS. For instance, there are age and gender differentials associated with HGS^18–21^. Low height^22–24^, insufficient weight^22,24,25^, a low BMI^19^, a sedentary lifestyle, and lower levels of physical activity and social participation^22,26–28^ are risk factors for low HGS. Further, research finds that chronic conditions, poor perceived health^25,29–32^, depressive symptoms, and insufficient sleep are significantly and inversely associated with HGS^33,34^. Worth noting is the possibility of reverse causality between some of these factors and HGS^35^. For example, while depressive symptoms can weaken muscle strength, weakened physical function is known to trigger depression^36^.

What remains indisputable is that research on HGS has increasingly gained attention. It is also clear that SES disparities exist in HGS^12,29^, and there are SES differentials in demographic, social, and behavioral factors mentioned above. These demographic, social, and behavioral factors likely contribute to the SES-related inequalities in declining HGS among older adults. An empirical investigation into this, however, is currently lacking. We are especially aware of no study within the Indian context that has assessed factors contributing to SES inequality in HGS among older adults.

Such an inquiry is important because, though population aging is happening worldwide (and India is not exempt from this demographic shift), aging in LMICs like India remains characteristically different from the experience of aging in high-income nations of the world. For one, the prevalence of muscle loss and weak muscle strength appears higher in most LMICs, like India, compared to high-income nations^37^. Two, and concomitantly, the social insurance systems, including long-term care options for older Indians, are considerably weaker than those available to their European and American counterparts. As a consequence, older Indians with compromised physical function may not receive the timely, adequate, and stable means of formal support that are available to their peers in high-income nations. This, in turn, would mean an increased economic and emotional burden on individuals, families, and both formal and informal systems of care.

Considering this, the present study aims to assess the SES and demographic, social, and behavioral factors associated with HGS among older adults and the contribution of those factors to the concentration of weak HGS among older adults belonging to lower SES. An inquiry of this nature would help policymakers and practitioners focus on those older adults who are susceptible to physical decline and frailty.

## Methods

### Study sample

**Figure 1:**
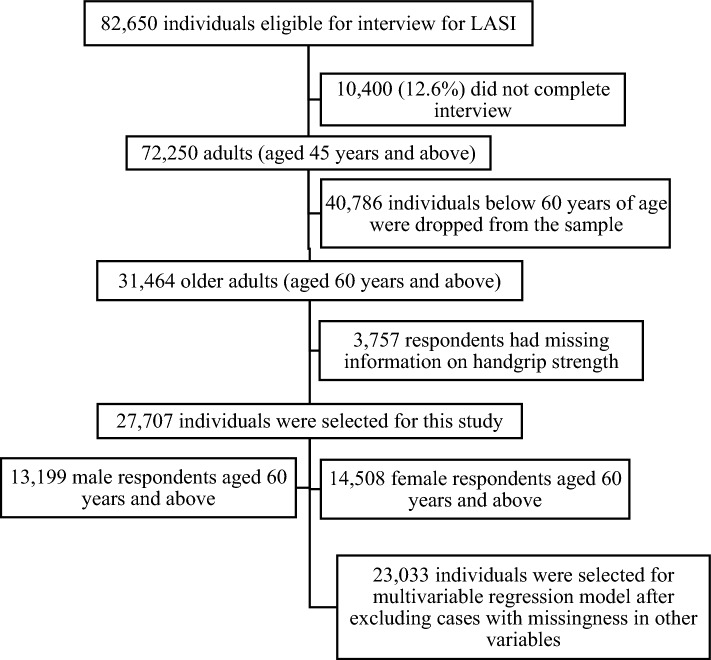
Sample selection criteria for this study.

Data come from wave 1 (2017–2018) of the Longitudinal Ageing Study in India (LASI). LASI is a nationally representative survey of 72,250 adults aged 45 and above across all states and union territories of India^38^. The LASI survey adopted a multistage stratified area probability cluster sampling design with a three-stage sampling design in rural areas and a four-stage sampling design in urban areas. The detailed methodology, with complete information on the survey design and data collection, was published in the survey report^38^. The present study is conducted on older respondents aged 60 years and above. Thus, after excluding the missing cases for the outcome variable of grip strength (n = 3757), the sample for the present study consisted of 27,707 older adults aged 60 years and older. In the multivariable analysis (Fig. 1), adjusting for variables that have missingness (cognitive impairment, n = 5289; body mass index, n = 3659; and depression, n = 812) yielded an analytical sample of 23,033 older adults aged 60 and above.

### Variable measurements

#### Handgrip strength (outcome variable)

The LASI survey assessed HGS in kilograms for both hands by trained research assistants using a handheld Smedley's Hand Dynamometer. Subjects were instructed to sit in a chair with arm support to rule out gravitational force. Further, the subjects were asked to press the dynamometer three times in each hand, and the maximum of the six values was chosen as the grip strength. Health investigators collected two readings of grip strength for both hands (dominant and non-dominant). The investigator says the following to the respondent “Now I would like to assess the strength of your hand in a gripping action. I will ask you to squeeze this handle as hard as you can, just for a couple of seconds and then let go. I will take alternately two measurements from your right and your left hand. Begin the test with the left hand. Before we begin, I would like to make sure it is safe for you to do this measurement. Have you had surgery or experienced any swelling, inflammation, severe pain, or injury in one or both hands within the last 6 months?” Take 30 s-rests between two measurements. Record measurements to the nearest 0.5 kg in the table below.

The final HGS score (in kg) was calculated as the maximum grip strength value of the dominant hand, along with an average score (in kg) of two successive trials in that hand^38^. Since standards based on high-income countries pose a considerable problem in properly assessing grip strength, the reference standards for phenotypically different Indian populations were used in the current study. Thus, the cut-off for weak HGS for males was 19.5 kg; for females, it was 12.5 kg, taken as the lowest 25th percentile or lowest quartile, validated in the Indian context^39–41^. Sensitivity analysis was conducted using a cut-off suggested by the Asian Working Group on Sarcopenia, with less than 28 kg for males and less than 18 kg for females^42^.

#### Socioeconomic status variables

SES was measured using respondents’ educational status, paid work status, and household wealth. Educational status was coded as no formal education, primary, secondary, and higher. Paid work status was coded as "never worked", "currently working", "currently not working", and "retired". Based on recommendations for "better" indicators of SES in LMICs^43^, the monthly per capita consumption expenditure (MPCE) quintile was assessed using household consumption data. Sets of 11 and 29 questions on the expenditures on food and non-food items, respectively, were used to canvass the sample households. Food and non-food expenditures have been standardized for the 30-day reference period. The MPCE is computed and used as the summary measure of consumption^38^. The variable is then divided into five quintiles, i.e., from poorest to richest.

Further, for the inequality analysis, the wealth index was calculated using variables related to household assets, amenities, and housing quality. For constructing the wealth index in the LASI, we followed a similar approach used in the Demographic Health Surveys (DHS)^44^. We utilized a set of 46 variables that covers the broad domains of the household's wealth and amenities and access to financial institutions. Principal component analysis (PCA) was conducted to construct the composite wealth index. We observed that the first principal component with an Eigenvalue of 7.2 explained around 16 percent of the variance^45^. The factor scores of the variables were used as the weights in constructing the overall composite index. The details of the measurement are published elsewhere^46^. The five wealth quintiles were derived from the composite score: poorest, poorer, middle, richer, and richest.

In addition to objective markers of SES, the present study also considered the relevance of subjective social status for HGS and the extent of its contribution to SES-related inequity in HGS. Subjective social status was assessed using the Macarthur scale^47^ with a ladder technique, and the question used to assess the variable was, "Think of the ladder with ten stairs as representing where people stand in our society." At the top of the ladder are the people who are best off—those who have the most money, the most education, and the best jobs. At the bottom are the people who are the worst off—those who have the least money, the least education, and the worst jobs or no jobs. "The higher up you are on this ladder, the closer you are to the people at the very top, and the lower you are, the closer you are to the people at the very bottom of your society"^38^. The scale is used to measure subjective SES across different populations in India and other countries^48–50^. A score of 0–10 was generated per the number of rungs marked by the respondents and was dichotomized as "high", representing those who scored 6 and above, and otherwise "low".

#### Sociodemographic variables

Age was categorized into the groups of 60–69 years, 70–79 years, and 80+ years; living arrangement was recoded as living alone, living with spouse, living with children and living with others; marital status was recoded as currently married, widowed and others (others included divorced/separated/never married)^51^; work status was recoded as never worked, currently working, currently not working and retired^52^.

#### Health variables

Multimorbidity was assessed using the self-reported diagnosis of two or more chronic conditions by a health professional, and the conditions include hypertension, diabetes, heart disease, stroke, lung disease, cancer, bone-related disease, and psychiatric disorders. Poor self-rated health (SRH) was coded as yes and no. SRH was available available on a scale of five, representing good (very good, good, fair) and poor (poor and very poor).

Difficulty in activities of daily living (ADL) included having any difficulty with the following six activities: (a) walking across a room, (b) dressing, (c) bathing, (d) eating, (e) getting in and out of bed, and (f) toileting. Responses for the six items (1 = *yes*; 0 = *no*) were summed (range: 0–6). Older individuals who struggled with any activity for more than three months were labeled “having difficulties” and otherwise “no.” We included ADLs, given that difficulty in everyday functioning and independence can be crucial for life satisfaction.

Difficulty in instrumental activities of daily living (IADL) was assessed by asking respondents to indicate the difficulty they encounter when performing the following seven activities: grocery shopping, preparing meals, making phone calls, taking medication, doing household chores, managing finances, and getting oneself to an otherwise unfamiliar location^38^. Each item response was coded as 0 for “no difficulty” or 1 for “any difficulty”. Those who reported trouble with any of these activities for more than three months were labeled “having difficulty.” Otherwise, they were categorized as having “no difficulty.” Even though IADLs may not require hands-on assistance, difficulty in executing IADLs may compromise independent living, ultimately affecting life satisfaction.

Major depression among older adults with symptoms of dysphoria was calculated using the Short Form Composite International Diagnostic Interview (CIDI-SF) with a cut-off score of 3 or more on a scale of 0–10. This scale estimates a probable psychiatric diagnosis of major depression, has been validated in field settings, and is widely used in population-based health surveys^38^. Depression in this study was coded as 0 for “not having depression” and 1 for “having depression.”

Cognitive dysfunction was assessed using the items relating to memory, arithmetic, and executive functions, adapted from the Mini-Mental State Examination (MMSE), and the cognitive modules of the Health and Retirement Study, the China Health and Retirement Longitudinal Study (CHARLS), and the Mexican Health and Aging Study (MHAS). A composite cognitive score ranged between 0 and 43, and a higher score indicated better cognitive functioning. The respondents falling in the lowest percentile were considered to have cognitive dysfunction^50^.

#### Behavioral variables

Given the relevance of SES to behavioral factors and the importance of behavioral factors to health, we considered living arrangements (with spouse and child, living alone, with spouse, with child, and others), BMI (underweight, normal weight, overweight, obese), chewing tobacco (no, yes), smoking tobacco (no, yes), and alcohol consumption (no, yes).

Further, vigorous physical activity included activities such as running or jogging, swimming, going to a health center or gym, cycling, or digging with a spade or shovel, heavy lifting, chopping, farm work, fast bicycling, and cycling with loads^38^. Moderate physical activity included activities such as cleaning the house, washing clothes by hand, fetching water or wood, drawing water from a well, gardening, bicycling at a regular pace, walking at a moderate pace, dancing, doing floor or stretching exercises^38^. Similarly, yoga-related activities were assessed using the survey question, “How often do you engage in activities such as yoga, meditation, asana, pranayama, or similar?”^38^. Those who reported any of the respective activities at least once a month (responses were every day, more than once a week, once a week, one to three times a month, and hardly ever or never) were categorized into yes and otherwise no.

#### Other covariates

Religion was coded as Hindu, Muslim, Christian, and Others. The respondent’s self-reported social group is coded as Scheduled Tribe (ST), Scheduled Caste (SC), Other Backward Class (OBC), and Others^53^. Place of residence was coded as (rural and urban). The regions were coded as North, Central, East, Northeast, West, and South.

### Statistical approach

The present study used descriptive statistics, bivariate, and multivariable analyses, and decomposition techniques to accomplish the study objectives. The bivariate analysis was used to present the weighted percentage distribution of the weak HGS with the background characteristics of the respondents. The Chi-Square test of independence is used to determine if there is a significant relationship between variables. Multivariable logistic regression was applied to determine what factors were linked to weak HGS. To adjust the effect of complex survey design (sample weights, strata, and clustering), the “svy” command was employed in STATA. The results from the multivariable analysis are presented as crude and adjusted odds ratios (adjusted for all the selected covariates) with 95% confidence intervals. Variance inflation factor (VIF) was generated in Stata to check the multicollinearity and there was no evidence of multicollinearity in the variables used.

Further, the concentration curve and index (CCI) were used to determine the inequalities in the prevalence of weak HGS by wealth index scores. The curve depicts how the cumulative shares of weak HGS (y-axis) are accounted for by the cumulative percentage of the individuals ranked by wealth scores (x-axis)^54^. If all the individuals had the exact prevalence of weak HGS, regardless of their wealth status, the curve would be a 45° line from the lower-left corner to the upper-right corner, also known as the "line of equality." Conversely, if weak HGS were more prevalent among poorer people, the curve would lie above the "line of equality" and vice versa. The farther the curve is away from the baseline, represented by the equality line, the more unequal the distribution of the prevalence of weak HGS^55^. The CCI corresponds to twice the area between the curve and the line of equality^56^.

#### Decomposition of the concentration index

The present study used Wagstaff's decomposition approach to reveal the contribution of each explanatory variable to the measured outcome inequality (weak HGS)^57^. According to Wagstaff, a linear regression model linking the outcome variable (y) to a set of k explanatory variables (${{\text{x}}}_{{\text{k}}}$) is:

1 $${{\text{y}}}_{{\text{i}}}={\upalpha }+{\sum }_{{\text{k}}}{\upbeta }_{{\text{k}}}{{\text{x}}}_{{\text{ki}}}+{\upvarepsilon }_{{\text{i}}}$$

where ${{\text{x}}}_{{\text{ki}}}$ is a set of *k* explanatory variables for the *i*th individual, ${\beta }_{k}$ signifies the coefficient, and ${\upvarepsilon }_{{\text{i}}}$ is an error term. Given the association of ${{\text{y}}}_{{\text{i}}}$ and ${{\text{x}}}_{{\text{ki}}}$, in Eq. (1), the concentration index for ${\text{y}}$, can be written as follows:

2 $${\text{C}}={\sum }_{{\text{k}}}(\frac{{\upbeta }_{{\text{k}}}{\overline{{\text{x}}} }_{{\text{k}}}}{\upmu }){{\text{C}}}_{{\text{k}}}+\frac{{{\text{GC}}}_{\upvarepsilon }}{\upmu }$$

where ${\text{C}}$ denotes the overall concentration index, $\upmu$ is the mean of ${\text{y}}$, ${\overline{{\text{x}}} }_{{\text{k}}}$ is the mean of ${{\text{x}}}_{{\text{k}}}$, ${{\text{C}}}_{{\text{k}}}$ is the normalized concentration index for ${{\text{x}}}_{{\text{k}}}$; $\frac{{\upbeta }_{{\text{k}}}{\overline{{\text{x}}} }_{{\text{k}}}}{\upmu }$ is the elasticity of health variable with the explanatory variables, and ${{\text{GC}}}_{\upvarepsilon }$ is the generalized concentration index for ${\varepsilon }_{i}$ (residual component). Equation (2) suggests that the concentration index consists of explained and residual (unexplained) components^54^. In most cases, the outcome variables are rarely continuous. We have approximated decomposition analysis by using marginal effects on the logit model. A linear approximation of the non-linear estimation can be represented as:

3 $${{\text{y}}}_{{\text{i}}}={{\upalpha }}^{{\text{m}}}+{\sum }_{{\text{k}}}{\upbeta }_{{\text{k}}}^{{\text{m}}}{{\text{x}}}_{{\text{ki}}}+{\upmu }_{{\text{i}}}$$

where ${\upbeta }_{{\text{k}}}^{{\text{m}}}$ is the marginal effects ($\frac{{\text{dy}}}{{\text{dx}}}$) of each x; ${\upmu }_{{\text{i}}}$ signifies the error term generated by the linear approximation. The concentration index for the outcome variable (y) (in our case, weak HGS) is given as:

4 $${\text{CI}}={\sum }_{{\text{k}}}(\frac{{\upbeta }_{{\text{k}}}{\overline{{\text{x}}} }_{{\text{k}}}}{\upmu }){{\text{C}}}_{{\text{k}}}+{{\text{GC}}}_{\upvarepsilon }/\upmu$$

where $\mu$ is the mean of y, ${\overline{x} }_{k}$ is the mean of ${x}_{k}$, ${C}_{k}$ is the concentration index for ${x}_{k}$ (defined analogously to C), and $G{C}_{\varepsilon }$ is the generalized concentration index for the error term ($\varepsilon )$. Equation (1) shows that C is equal to a weighted sum of the concentration indices of the k regressor, where the weight for ${x}_{k}$ is the elasticity of y with respect to ${x}_{k}$ $\left({\eta }_{k}= {\beta }_{k}\frac{{\overline{x} }_{k}}{\mu }\right)$. The residual component captured by the last term reflects the socioeconomic inequality in health that is not explained by systematic variation in the regressor by income, which should approach zero for a well-specified model. Each contribution is the product of elasticity with the degree of economic inequality. Moreover, the percentage contribution is obtained by dividing each absolute contribution by total absolute contribution multiplied by 100 to obtain the estimates.

The aim of this decomposition analysis was similar to the mediation analysis to quantify the percentage of the total effect mediated by each factor in the model. The first two columns show the elasticities and CCI for each predictor. The rest of the columns show each predictor's absolute contributions and total percentage contributions to economic inequalities in successful ageing. The value of the absolute contribution depicts the extent of inequality contributed by a particular explanatory variable.

During the multivariable analysis, the observations with missing information in any of the study variables (n = 8453) were dropped and the final study sample was 23,011 older adults. The socio-demographic characteristics such as age, sex, education and household wealth quintiles of the included and excluded samples were compared. We observed no statistically significant differences in the two samples, suggesting no potential impact of missingness in the current analyses. Also, to examine the potential impact of the inclusion of individuals aged 80 and above (who may represent a selective group of healthier and longer-lived individuals) on our findings, we conducted a sensitivity analysis after excluding the 80+ age group.

### Statement of ethics and consent to participate

Ethics approval was obtained from the Central Ethics Committee on Human Research (CECHR) under the Indian Council of Medical Research (ICMR). And all methods were carried out in accordance with the relevant guidelines and regulations of ICMR. The survey agencies that conducted the field survey for the data collection have collected prior informed consent (signed and oral) for both the interviews and biomarker tests from the eligible respondents in accordance with Human Subjects Protection. Informed consent was obtained from all subjects and/or their legal guardian(s).

## Results

### Profile of the study sample and the prevalence of better/weaker HGS

Table 1 presents the sample characteristics and the prevalence estimates of better and weaker HGS—a proportion of 10.3% of the sample population aged 80 or above in this study. A slightly higher percentage of the sample was female (52.36% vs. 47.64%). Around 36% of the participants were widowed, and more than 56% of the sample had no formal education. Notably, 26.3% of the participants were underweight, whereas 16.3% were overweight and 5.4% were obese in this study. A higher percentage of the sample population belonged to rural areas (71.7%) than urban areas (28.3%).

**Table 1:** Sample characteristics and prevalence estimates of better and weak hand grip strength.

Variable	Categories	Sample distribution	Better hand grip strength	Weak hand grip strength	Chi-square test p-value
		Frequency (col w%)	Row w%	Row w%	
Age	60–69 years	17,008 (59.99)	15,687 (92.41)	1321 (7.59)	< 0.001
	70–79 years	8002 (29.71)	7329 (91.7)	673 (8.3)	
	80 years & above	2697 (10.3)	2178 (80.79)	519 (19.21)	
Sex	Male	13,402 (47.64)	11,370 (84.45)	2032 (15.55)	< 0.001
	Female	14,305 (52.36)	13,824 (96.96)	481 (3.04)	
Marital status	Married	17,764 (62.23)	16,055 (90.15)	1709 (9.85)	< 0.001
	Widowed	9230 (35.59)	8508 (92.73)	722 (7.27)	
	Divorced and others	713 (2.18)	631 (87.06)	82 (12.94)	
Education	No	14,788 (56.44)	13,498 (91.49)	1290 (8.51)	< 0.001
	Primary	5219 (17.72)	4667 (89.68)	552 (10.32)	
	Secondary	5431 (18.15)	4933 (91.17)	498 (8.83)	
	Higher	2269 (7.69)	2096 (90.07)	173 (9.93)	
Work status	Never worked	7632 (26.21)	7311 (96.63)	321 (3.37)	< 0.001
	Not working	9547 (35.57)	8224 (86.41)	1323 (13.59)	
	Working	8154 (30.96)	7516 (92.15)	638 (7.85)	
	Retired	2374 (7.26)	2143 (88.28)	231 (11.72)	
Alcohol	No	25,849 (94.54)	23,610 (91.39)	2239 (8.61)	< 0.001
	Yes	1826 (5.37)	1556 (84.26)	270 (15.74)	
Smoking	No	23,024 (84.21)	21,136 (91.83)	1888 (8.17)	< 0.001
	Yes	4648 (15.71)	4028 (86.6)	620 (13.4)	
Chewing tobacco	No	21,365 (74.9)	19,456 (91.32)	1909 (8.68)	0.172
	Yes	6307 (25.02)	5708 (90.09)	599 (9.91)	
Vigorous activity	No	18,960 (67.69)	17,137 (90.63)	1823 (9.37)	< 0.001
	Yes	8712 (32.22)	8029 (91.83)	683 (8.17)	
Moderate activity	No	25,002 (89.76)	22,662 (90.78)	2340 (9.22)	< 0.001
	Yes	2547 (9.64)	2389 (93.08)	158 (6.92)	
Yoga-related activity	No	23,366 (85.84)	21,088 (90.41)	2278 (9.59)	< 0.001
	Yes	4292 (14.03)	4065 (94.67)	227 (5.33)	
Multimorbidity	No	20,908 (76.21)	19,084 (91.21)	1824 (8.79)	0.001
	Yes	6782 (23.74)	6096 (90.36)	686 (9.64)	
ADL difficulty	No	22,211 (77.92)	20,463 (92.45)	1748 (7.55)	< 0.001
	Yes	5485 (22.05)	4721 (85.88)	764 (14.12)	
IADL difficulty	No	15,795 (52.82)	14,687 (92.98)	1108 (7.02)	< 0.001
	Yes	11,861 (47.09)	10,461 (88.77)	1400 (11.23)	
Poor SRH	No	21,425 (76.21)	19,744 (92.2)	1681 (7.8)	< 0.001
	Yes	6266 (23.75)	5436 (87.16)	830 (12.84)	
Depression	No	25,747 (91.4)	23,447 (90.99)	2300 (9.01)	0.005
	Yes	1928 (8.52)	1719 (91.2)	209 (8.8)	
Cognitive dysfunction	No	20,831 (75.1)	19,131 (91.67)	1700 (8.33)	< 0.001
	Yes	2918 (11.28)	2590 (90.08)	328 (9.92)	
	Missing	3958 (13.62)			
BMI	Normal	14,213 (50.11)	12,990 (91.8)	1223 (8.2)	< 0.001
	Underweight	6336 (26.3)	5503 (87.35)	833 (12.65)	
	Overweight	5004 (16.26)	4727 (94.05)	277 (5.95)	
	Obese	1588 (5.41)	1515 (95.6)	73 (4.4)	
	Missing	566 (1.92)			
Wealth quintiles (MPCE)	Poorest	5656 (21.58)	5113 (91.24)	543 (8.76)	0.047
	Poor	5725 (21.67)	5165 (90.7)	560 (9.3)	
	Middle	5711 (20.87)	5206 (90.52)	505 (9.48)	
	Rich	5460 (19.45)	4992 (91.35)	468 (8.65)	
	Richest	5155 (16.43)	4718 (91.29)	437 (8.71)	
Subjective social status	Low	14,862 (56.94)	13,389 (90.33)	1473 (9.67)	< 0.001
	High	12,522 (42.06)	11,529 (92.02)	993 (7.98)	
Social group	SC	4534 (18.92)	4075 (90.53)	459 (9.47)	< 0.001
	ST	4599 (8)	4203 (91.01)	396 (8.99)	
	OBC	10,537 (45.73)	9427 (90.13)	1110 (9.87)	
	Others	8037 (27.35)	7489 (92.77)	548 (7.23)	
Place of residence	Urban	9239 (28.29)	8535 (92.26)	704 (7.74)	< 0.001
	Rural	18,468 (71.71)	16,659 (90.5)	1809 (9.5)	
Religion	Hindu	20,268 (82.65)	18,351 (90.8)	1917 (9.2)	< 0.001
	Muslim	3287 (10.93)	3005 (92.94)	282 (7.06)	
	Christian	2800 (2.8)	2565 (87.32)	235 (12.68)	
	Others	1352 (3.62)	1273 (92.64)	79 (7.36)	
Region	North	5141 (12.89)	4881 (94.41)	260 (5.59)	< 0.001
	Central	3754 (21.13)	3518 (94.7)	236 (5.3)	
	East	5235 (24.43)	4776 (91.49)	459 (8.51)	
	North-east	3320 (2.94)	3134 (95.27)	186 (4.73)	
	West	6553 (22.1)	5535 (85.1)	1018 (14.9)	
	South	3704 (16.5)	3350 (90.02)	354 (9.98)	
Total		27,707 (100)	25,194 (91)	2513 (9)	

*ADL* activities of daily living, *IADL* instrumental activities of daily living, *SRH* self-rated health, *BMI* Body mass index, *MPCE* Monthly per capita consumption expenditure.

Around 9% of older adults in this study had a weak HGS. Weak HGS was significantly higher among those who were 80 years of age or older (19.21%), males (15.55%), those who were currently not working (13.59%), those who consumed alcohol (15.74%), smoked (13.4%), and those who had ADL (14.12%) or IADL difficulty (11.23%).

### Multivariable logistic estimates of weak HGS among older adults

Logistic regression estimates of weak HGS are presented in Table 2. Older adults who were 80 or older had higher odds of having weak HGS [OR: 1.73, CI 1.25–2.40] than those who were 60–69. Older women had lower odds of having weak HGS [OR: 0.09, CI 0.07–0.12] than their male counterparts. Those respondents with a secondary level of education had lower odds of having weak HGS [OR: 0.76, CI 0.61–0.96] compared to peers with no formal education. Smoking and yoga-related activities were protective against having weak HGS; older adults who smoked [OR: 0.74, CI 0.60–0.90] or engaged in yoga-related activities [OR: 0.68, CI 0.51–0.89] had lower odds of having weak HGS compared to their respective counterparts. Health variables such as difficulty in ADL [OR: 1.48, CI 1.19–1.84], difficulty in IADL [OR: 1.43, CI 1.19–1.70], being underweight [OR: 1.59, CI 1.34–1.89], and having cognitive impairment [OR: 1.34, CI 1.05–1.70] were found to be risk factors for having weak HGS. Further, belonging to the richest wealth quintile [OR: 0.74, CI 0.54–0.99] and having a higher subjective social status [OR: 0.82, CI 0.70–0.97] were associated with lower odds of having weak HGS.

**Table 2:** Multivariable logistic regression estimates of weak grip strength by background variables among older adults, LASI-2017-18.

Variable	Categories	Crude odds ratio (95% CI)	Adjusted odds ratio (95% CI)
Age	60–69 years	Ref	Ref
	70–79 years	1.10 (0.96–1.27)	0.72*** (0.60–0.85)
	80 years & above	2.90*** (2.32–3.61)	1.73** (1.25–2.40)
Sex	Male	Ref	Ref
	Female	0.17*** (0.15–0.20)	0.09*** (0.07–0.12)
Marital status	Married	Ref	Ref
	Widowed	0.72*** (0.62–0.83)	1.16 (0.95–1.42)
	Divorced and others	1.36 (0.92–2.01)	0.83 (0.51–1.36)
Education	No	Ref	Ref
	Primary	1.24*** (1.06–1.44)	0.81 (0.65–1.01)
	Secondary	1.04 (0.87–1.24)	0.76* (0.61–0.96)
	Higher	1.19 (0.79–1.79)	0.99 (0.68–1.43)
Work status	Never worked	Ref	Ref
	Not working	4.51*** (3.70–5.50)	1.32 (0.99–1.76)
	Working	2.44*** (1.98–3.02)	0.84 (0.62–1.14)
	Retired	3.80*** (2.54–5.70)	1.27 (0.86–1.86)
Alcohol	No	Ref	Ref
	Yes	1.98*** (1.60–2.45)	1.06 (0.80–1.41)
Smoking	No	Ref	Ref
	Yes	1.74*** (1.50–2.02)	0.74** (0.60–0.90)
Chewing tobacco	No	Ref	Ref
	Yes	1.16** (1.01–1.33)	0.84 (0.69–1.02)
Vigorous activity	No	Ref	Ref
	Yes	0.86* (0.73–1.02)	0.9 (0.74–1.09)
Moderate activity	No	Ref	Ref
	Yes	0.73 (0.46–1.16)	0.83 (0.57–1.20)
Yoga-related activity	No	Ref	Ref
	Yes	0.53*** (0.44–0.64)	0.68** (0.51–0.89)
Multimorbidity	No	Ref	Ref
	Yes	1.11 (0.95–1.29)	0.91 (0.73–1.14)
ADL difficulty	No	Ref	Ref
	Yes	2.01*** (1.72–2.36)	1.48*** (1.19–1.84)
IADL difficulty	No	Ref	Ref
	Yes	1.68*** (1.46–1.92)	1.43*** (1.19–1.70)
Poor SRH	No	Ref	Ref
	Yes	1.74*** (1.51–2.00)	1.2 (0.99–1.47)
Depression	No	Ref	Ref
	Yes	0.97 (0.79–1.20)	0.82 (0.62–1.09)
Cognitive dysfunction	No	Ref	Ref
	Yes	1.21** (1.01–1.46)	1.34* (1.05–1.70)
BMI	Normal	Ref	Ref
	Underweight	1.62*** (1.42–1.86)	1.59*** (1.34–1.89)
	Overweight	0.71* (0.50–1.00)	0.73* (0.55–0.98)
	Obese	0.52*** (0.35–0.75)	0.84 (0.53–1.31)
Wealth quintiles (MPCE)	Poorest	Ref	Ref
	Poor	1.07 (0.90–1.28)	0.92 (0.74–1.15)
	Middle	1.09 (0.86–1.38)	1.06 (0.84–1.33)
	Rich	0.99 (0.81–1.20)	0.9 (0.69–1.17)
	Richest	0.99 (0.81–1.22)	0.74* (0.54–0.99)
Subjective social status	Low	Ref	Ref
	High	0.81*** (0.70–0.94)	0.82* (0.70–0.97)
Social group	SC	Ref	Ref
	ST	0.94 (0.75–1.19)	0.87 (0.63–1.19)
	OBC	1.05 (0.88–1.24)	0.91 (0.74–1.10)
	Others	0.75*** (0.62–0.90)	0.91 (0.71–1.16)
Place of residence	Urban	Ref	Ref
	Rural	1.25** (1.03–1.52)	0.74* (0.57–0.96)
Religion	Hindu	Ref	Ref
	Muslim	0.75*** (0.61–0.92)	1 (0.68–1.49)
	Christian	1.43** (1.07–1.92)	0.84 (0.54–1.32)
	Others	0.78 (0.55–1.11)	0.88 (0.72–1.08)
Region	North	Ref	Ref
	Central	0.95 (0.75–1.18)	0.65** (0.48–0.87)
	East	1.57*** (1.28–1.93)	1.22 (0.92–1.62)
	North-east	0.84 (0.64–1.11)	0.72 (0.50–1.04)
	West	2.96*** (2.39–3.66)	2.93*** (2.26–3.80)
	South	1.87*** (1.50–2.35)	1.76*** (1.31–2.37)

*ADL* activities of daily living, *IADL* instrumental activities of daily living, *SRH* self-rated health, *BMI* Body mass index, *MPCE* monthly per capita consumption expenditure.

### Sensitivity analyses

Using the same cut-off point for older men and women may potentially introduce bias into the results and therefore, we conducted the multivariable analysis of the 25th percentile of HGS, separately for men and women, and yielded similar results (Table S1). The results were similar for men and women except for smoking (unlike men, women who smoked had higher odds of weak HGS). Given that life expectancy at 60 for female and male Indians is approximately 78–80 and 77–78 years, respectively^58^, individuals aged 80 and above may represent a selective group of healthier and longer-lived individuals. To ensure the robustness of your results, we conducted a sensitivity analysis by excluding this age group and observed similar results (Table S2). The sensitivity analysis using a different cut-off for weak HGS, suggested by the Asian Working Group on Sarcopenia, also indicated similar results (Table S3).

### Concentration index representing the wealth-based inequalities in weak HGS

**Figure 2:**
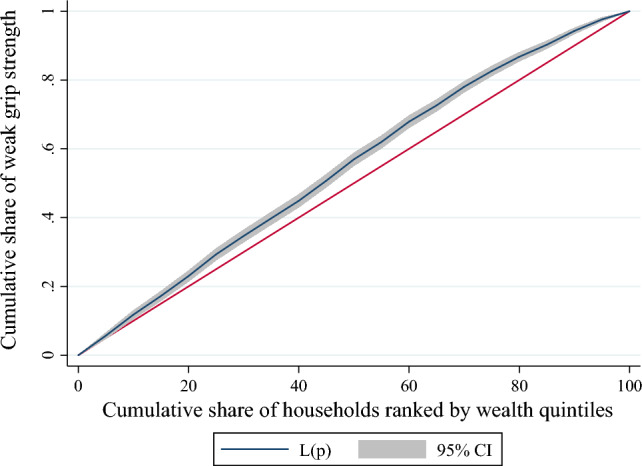
Concentration curve of the weak grip strength by household wealth quintiles.

Figure 2 reveals weak HGS among older adults from poor socioeconomic strata. The value of the concentration index is -0.05 (p < 0.001), which also confirms that the observed inequality in weak grip strength is significantly higher.

### Decomposition estimates of factors contributing to wealth-based inequalities in weak HGS

Table 3 represents the decomposition estimates of wealth-based inequality in weak HGS among older adults in India. The CCI indicates concentration index and negative CCI denotes that weak HGS was concentrated among poor older adults for that particular predictor and vice-versa. The absolute contribution is the product of elasticity and CCI whereas the percentage contribution is the proportion of absolute contribution multiplied by 100. The percentage contributions are a mixture of positives and negatives, which sum up to 100. Positive (negative) contributions of association can be interpreted by indicating that the total health inequality would be lower (higher) if that association had no impact on the health outcome. It was found that a higher percentage of the wealth-based inequality in weak HGS (concentrated among older adults from poor households) was explained by being underweight (38.83%), belonging to the richest wealth quintile (27.95%), and having a higher subjective social status (32.20%). Moreover, about 23.29% of the inequality in weak HGS was explained by Western region and 22.54% by female gender. Additionally, having a secondary level of education explained a higher percentage (22.09%) of inequality, followed by current working status (-20.68%). Rural residence (13.08%), having difficulty in IADL (12.21%), and engagement in yoga-related activities (11.55%) explained a higher percentage of wealth-based inequalities.

**Table 3:** Decomposition of the determinants of socioeconomic inequality in weak grip strength.

Variable	Categories	Elasticity	CCI	Contribution	Percent contribution
Age	60–69 years				
	70–79 years	− 0.0079	− 0.0205	0.00016	− 2.57
	80 years & above	0.0068	0.0062	0.00004	− 0.67
Sex	Male				
	Female	− 0.0812	− 0.0175	0.00142	− 22.54
Marital status	Married				
	Widowed	0.0004	− 0.0423	− 0.00002	0.27
	Divorced and others	− 0.0001	− 0.0587	0.00001	− 0.09
Education	No				
	Primary	− 0.0034	0.0501	− 0.00017	2.70
	Secondary	− 0.0054	0.2579	− 0.00139	22.09
	Higher	− 0.0011	0.6031	− 0.00066	10.52
Work status	Never worked				
	Not working	0.0041	− 0.0791	− 0.00032	5.14
	Working	− 0.0096	− 0.1358	0.00130	− 20.68
	Retired	− 0.0004	0.4785	− 0.00019	3.04
Alcohol	No				
	Yes	0.0009	− 0.1481	− 0.00013	2.11
Smoking	No				
	Yes	− 0.004	− 0.0419	0.00017	− 2.66
Chewing tobacco	No				
	Yes	− 0.0036	− 0.1395	0.00050	− 7.96
Vigorous activity	No				
	Yes	− 0.003	− 0.0563	0.00017	− 2.68
Moderate activity	No				
	Yes	− 0.0011	0.2961	− 0.00033	5.17
Yoga-related activity	No				
	Yes	− 0.0028	0.2602	− 0.00073	11.55
Multimorbidity	No				
	Yes	− 0.0018	0.1804	− 0.00032	5.15
ADL difficulty	No				
	Yes	0.0074	− 0.068	− 0.00050	7.98
IADL difficulty	No				
	Yes	0.0121	− 0.0636	− 0.00077	12.21
Poor SRH	No				
	Yes	0.0043	− 0.0808	− 0.00035	5.51
Depression	No				
	Yes	− 0.001	− 0.1642	0.00016	− 2.60
Cognitive dysfunction	No				
	Yes	0.0018	− 0.2738	− 0.00049	7.82
BMI	Normal				
	Underweight	0.0092	− 0.2661	− 0.00245	38.83
	Overweight	− 0.0025	0.2646	− 0.00066	10.49
	Obese	− 0.0005	0.4351	− 0.00022	3.45
Wealth quintiles (MPCE)	Poorest				
	Poor	− 0.0012	− 0.2616	0.00031	− 4.98
	Middle	0.0005	0.1185	0.00006	− 0.94
	Rich	− 0.0013	0.4737	− 0.00062	9.77
	Richest	− 0.0025	0.812	− 0.00203	32.20
Subjective social status	Low				
	High	− 0.0068	0.2592	− 0.00176	27.95
Social group	SC				
	ST	− 0.0007	− 0.3242	0.00023	− 3.60
	OBC	− 0.0032	0.0041	− 0.00001	0.21
	Others	− 0.0008	0.2073	− 0.00017	2.63
Place of residence	Urban				
	Rural	− 0.0054	− 0.1527	0.00082	− 13.08
Religion	Hindu				
	Muslim	− 0.0028	− 0.0596	0.00017	− 2.65
	Christian	0.0002	0.0186	0.00000	− 0.06
	Others	− 0.0002	0.2339	− 0.00005	0.74
Region	North				
	Central	− 0.0051	− 0.1583	0.00081	− 12.80
	East	0.0023	− 0.1569	− 0.00036	5.72
	North-east	− 0.0004	0.0367	− 0.00001	0.23
	West	0.0194	0.0757	0.00147	− 23.29
	South	0.0066	0.092	0.00061	− 9.63
Total				− 0.00631	100.00
Actual CCI				− 0.05438	
Residual CCI				− 0.04807	

*CCI* concentration index, *ADL* instrumental activities of daily living, *ADL* activities of daily living, *SRH* self-rated health, *BMI* body mass index, *MPCE* monthly per capita consumption expenditure.

## Discussion

The purpose of this study was to assess, using the CCI, the extent of SES-related inequality in HGS. As expected and comparable to previous studies^12,29^, the CCI for HGS in our study unveiled an uneven distribution of HGS among older adults in India. Our findings reveal statistically consequential SES-related disparities that benefit those with higher SES, with weak HGS concentrated in older Indians of lower socioeconomic standing. We also explored the contribution of several demographic, social, and behavioral factors to SES-related disparities in HGS. Our findings show that being female, underweight, having a secondary level of education, current employment, belonging to the richest wealth quintile, higher subjective social status, IADL limitations, and rural residence contribute positively or negatively to SES-related inequalities in HGS.

Consistent with previous studies, we found older adults aged 80 and above to have higher odds of weak HGS than peers aged 60–69 years. Typically, HGS peaks between ages 30 and 40 and continues to dwindle with advancing age in both women and men^59–62^. Although men report higher HGS than women, their grip strength usually declines faster with age than women's, and the wider gender difference found earlier in the life course tends to narrow with age^59–63^. This may partly explain our finding that older women report lower odds of weak HGS than their male counterparts. The observed male disadvantage in HGS could also be explained by the higher prevalence of cardiovascular conditions, multimorbidity, and body composition. Given sex differences in BMI and body composition, HGS should not be the unitary factor in gauging muscle strength or physical fitness^64,65^. Future studies are warranted to examine the gender differences in HGS using different cut-points based on BMI and other body composition measurements.

Gender also contributes to SES-related inequality in HGS, with older Indian men having higher odds of weak HGS relative to their older female peers. This is surprising given the otherwise well-documented male advantage in socioeconomic resources^66,67^ and men’s relatively lower exposure to stressful life events (e.g., intimate partner violence, reproductive health risks, workplace discrimination)^68^, both of which predict greater physical vitality. For one, it could be that older men of lower SES may have worked in physically hazardous environments with toxic contaminants and dangerous equipment^69–71^. Physical activity, in general, is positively associated with HGS^72^. However, chronic physical exertion or the wrong type of physical activity could damage to physical functioning, including muscle strength^73^. Two, for socioeconomically marginalized men, seeking health care may further challenge their masculinity and, in essence, their sense of self-worth. Relatedly, men often are resistant to health information given their desire to maintain control and display resilience^74^, and this could be particularly so among older men of lower SES. Lastly, for men of lower SES, the responsibility of being a provider may take precedence over personal health^70^.

In addition to age and gender, being underweight was a factor significantly linked with weak HGS. Add to that, it was the most important contributor to SES-related inequity in HGS. This substantiates previous studies that point out a link between being underweight and weaker HGS in older age groups^75^. It also reflects the reality that a sizeable proportion of older men and women in India are underweight and undernourished^76^, which is often reflective of food insecurity and perhaps inadequate forms of formal and informal social support. Considering this and in light of the present study’s findings, we suggest that efforts to maintain muscle mass may significantly influence preserving HGS in older adults, especially among lower social groups.

Interestingly, among men, but not women, we found smokers to report lower odds of weak HGS than non-smokers. The finding of smoking associated with lower odds of weak HGS among men is contrary to empirical research that finds that smoking is positively associated with weaker HGS^77^ and overall physical frailty^78^. Non-smokers may have smoked in the past and desisted smoking due to health problems (e.g., lung and respiratory complications), which may manifest in the weaker HGS in this group. Our finding matches one recent study by Kim et al.^79^, where smokers and ex-smokers reported higher mean HGS than nonsmokers. One study revealed a more nuanced finding where current smoking status was positively predictive of debility among respondents in their 1950s, but negatively linked with debility for respondents in their 1970s^80^. This may reflect two realities reported in research. Smoking is likely to affect muscle strength through several mechanisms, one being the increased level of carbon dioxide, which inhibits respiratory and muscle protein synthesis, rendering a higher risk of musculoskeletal injury^81,82^. Alternatively, the nicotine in tobacco smoke may stimulate an initial and instant beneficial effect on motor skills^82^. Considering these conflicting patterns, the overall health repercussions of smoking, and the fact that smoking is a modifiable factor, we recommend that future scholars use longitudinal studies to explore the relationship between trajectories of smoking and HGS among older Indians.

Another notable finding in this study is the significance of subjective social status for weak HGS and its contributing influence on SES-related inequality in HGS. This corroborates evidence finding an additional psychosocial element tied to SES influencing health and well-being, even after accounting for objective markers of SES^83–85^. Subjective social status captures the complexities associated not simply with one’s financial resources but one’s overall social and economic standing relative to others in their social network^84,86–88^. Many have argued that compared with objective SES, perceived social status is indicative of a stronger and more consistent association with psychological functioning, such as stress and negative emotions^83^, and health-related factors^83,89^, all of which negatively affect physical function. Otherwise stated, perceived social ranking within a social structure may “produce motivations, preferences, and opportunities”^90^, p.1 that shape health behaviors and, in turn, health outcomes, including HGS^83,90,91^.

Among health covariates, we found difficulty in ADL and IADL and cognitive impairment to emerge as risk factors for weak HGS. Individuals with ADL and IADL difficulties may be limited in the extent to which they remain physically active. Inadequate physical activity, in turn, can weaken HGS, as shown in existing studies^92^. Resistance training, which is an effective tool for building muscle strength, should be encouraged among those with difficulty in ADLs, and specific attention should be given to lower SES older adults who may have fewer structured opportunities to engage in physical activity.

Like ADL and IADL limitations, we find that cognitive impairment is associated with weaker HGS. Yet again, it is possible that older adults with cognitive impairment may reduce physical activity (e.g., exercise), that is essential for maintaining muscle strength^93–95^. Additionally, the association between cognitive deficits and weak HGS may signal the existence of shared covariance (e.g., decreased levels of sex steroids and increased inflammation) that may negatively affect both cognitive and muscle functioning^93^. For example, some studies find that high blood levels of interleukin-6 (a pro-inflammatory cytokine) are significantly linked with weaker HGS and cognitive decline, even after considering relevant confounders^93,96,97^. Subsequent waves of LASI data may enable future research to unravel the intricate ties between types and intensity of physical activity, cognitive decline, and HGS. For instance, if the cognitive deterioration is accompanied by diminishing muscle-strengthening activities, decreased muscle mass and strength may compromise one’s ability to perform ADLs and IADLs and ultimately lead to functional disability^93^.

Lastly, secondary education and current working status are linked with better HGS among older adults of higher SES. In other words, a reasonable proportion of the HGS advantage enjoyed by higher SES older adults is accrued due to higher education and current employment. This coincides with earlier research finding a positive link between education and HGS^98–100^. In addition to its association with increased access to better-paying jobs and quality health care, higher education is linked to better nutrition^101^, more physical activity^102,103^, fewer instances of smoking, and moderate drinking^104,105^—all of which, separately and cumulatively, could render better muscle strength in older ages. Like education, paid work may mean more tangible resources needed to maintain physical health, including better housing, safer neighborhoods with well-lit spaces for walks, access to gyms for exercise, and health care. Apart from these tangible resources, paid work may also mean regular physical and social activity, which could ultimately benefit muscle health.

### Limitations

First, because the study is cross-sectional, we are unable to draw causal or even temporal inferences. For evaluating the causal and long-term associations between SES and behavioral, social, and demographic components that affect HGS, future research will require longitudinal data with repeated assessments and time-varying covariates. Second, though HGS is a commonly used method of measuring muscle strength, additional measures of muscle potency, including the chair rise test, should be included in future research. Third, even with the wide range of covariates in our analysis, the problem of residual confounding resulting from unmeasured factors persists. For instance, while we account for working status, our study did not consider the nature of employment or the intensity of physical strain from one’s workplace conditions. Fourth, while we control for multimorbidity in our analyses, future studies should consider assessing, in addition to disease status, the nature, severity, and duration of chronic disease. Despite these limitations, our study has notable strengths. We used nationally representative data to estimate SES-related disparities in HGS among older Indians. Additionally, our study utilized a large sample size, and we relied on HGS, an objective health indicator of muscle strength and overall physical vitality in later life.

### Policy and practice implications

The broader implications of our findings are analogous to most social scientific research on SES disparities in health: to educate individuals to their full potential and redistribute income^106^ so that they have a fair shot at a healthy life and, in turn, healthy aging. However, given that such sweeping policy changes are politically intractable, there is the need for micro-level initiatives, such as publicly funded home-based nutritional and physical activity programs for underweight older adults and those with difficulty in ADL and IADL given that these factors contributed significantly to the SES disparities in HGS for older Indians. Likewise, given the significant link between cognitive deficits and weak HGS, trained volunteers can help cognitively impaired older adults with memory training techniques, such as rehearsal, organization, categorization, visualization, and the use of mnemonics^107^. Furthermore, physicians and other allied health professionals who wish to provide more individualized treatment to their socioeconomically vulnerable older patients will find it useful to know that a large portion of the SES inequality in HGS may be tied to lower "subjective" social status. The finding that, despite the otherwise well-documented male advantage in SES, older men endure higher odds of weak HGS than older women suggests that for interventions and therapeutics to work, providers and practitioners should consider multiple social and cultural contexts that may contribute to the differential health risks among older Indian men. There should be state-sponsored efforts to educate older adults on the importance of HGS, which is currently lacking in India, given that implementing practical and early interventions can prove crucial in protecting physical function and prolonging disability and dependence.

## Conclusion

The results of this study reveal significant SES-related inequalities in HGS, benefiting those of higher SES relative to their peers in poorer households. The study also reveals a variety of demographic, health, and behavioral factors contributing significantly to the SES-related inequities in older Indians. Social marketing campaigns for increasing physical function should consider the social and economic resources and constraints attached to structured and sustained physical activity. Further, given the evidence in the present study, public health policies and programs related to aging should be aimed at reducing social and economic inequities among older adults. Doing so would not only address questions of social justice, but it could also potentially alleviate the fiscal and emotional challenges associated with aging and old age.

### Supplementary Information

Supplementary Tables.

## Data Availability

The study uses secondary data which is available at the Gateway to Global Aging Data (https://g2aging.org/app/survey/get_data).

## References

[1] McGrath RP, Kraemer WJ, Snih SA, Peterson MD (2018). Handgrip strength and health in aging adults. Sports Med..

[2] Taekema DG, Gussekloo J, Maier AB, Westendorp RGJ, de Craen AJM (2010). Handgrip strength as a predictor of functional, psychological and social health. A prospective population-based study among the oldest old. Age Ageing..

[3] Bohannon RW (2008). Hand-grip dynamometry predicts future outcomes in aging adults. J. Geriatr. Phys. Ther..

[4] Bohannon RW (2019). Grip strength: An indispensable biomarker for older adults. Clin. Interv. Aging..

[5] López-Bueno R, Andersen LL, Calatayud J, Casaña J, Grabovac I, Oberndorfer M (2022). Associations of handgrip strength with all-cause and cancer mortality in older adults: A prospective cohort study in 28 countries. Age Ageing..

[6] Musalek C, Kirchengast S (2017). Grip strength as an indicator of health-related quality of life in old age—A pilot study. Int. J. Environ. Res. Public Health..

[7] Alfaro-Acha A, Snih SA, Raji MA, Kuo Y-F, Markides KS, Ottenbacher KJ (2006). Handgrip strength and cognitive decline in Older Mexican Americans. J. Gerontol. A Biol. Sci. Med. Sci..

[8] García-Hermoso A, Cavero-Redondo I, Ramírez-Vélez R, Ruiz JR, Ortega FB, Lee D-C (2018). Muscular strength as a predictor of all-cause mortality in an apparently healthy population: A systematic review and meta-analysis of data from approximately 2 million men and women. Arch. Phys. Med. Rehabil..

[9] Lee SY (2021). Handgrip strength: An irreplaceable indicator of muscle function. Ann. Rehabil. Med..

[10] Rijk JM, Roos PR, Deckx L, van den Akker M, Buntinx F (2016). Prognostic value of handgrip strength in people aged 60 years and older: A systematic review and meta-analysis. Geriatr. Gerontol. Int..

[11] Roberts HC, Denison HJ, Martin HJ, Patel HP, Syddall H, Cooper C (2011). A review of the measurement of grip strength in clinical and epidemiological studies: Towards a standardised approach. Age Ageing..

[12] Arokiasamy P, Selvamani Y, Jotheeswaran AT, Sadana R (2021). Socioeconomic differences in handgrip strength and its association with measures of intrinsic capacity among older adults in six middle-income countries. Sci. Rep..

[13] Leopold L, Engelhardt H (2013). Education and physical health trajectories in old age. Evidence from the Survey of Health, Ageing and Retirement in Europe (SHARE). Int. J. Public Health..

[14] Mohd Hairi F, Mackenbach JP, Andersen-Ranberg K, Avendano M (2010). Does socio-economic status predict grip strength in older Europeans? Results from the SHARE study in non-institutionalised men and women aged 50+. J. Epidemiol. Community Health..

[15] Pengpid S, Peltzer K (2018). Hand grip strength and its sociodemographic and health correlates among older adult men and women (50 years and older) in Indonesia. Curr. Gerontol. Geriatr. Res..

[16] Thorpe RJ, Simonsick E, Zonderman A, Evans MK (2016). Association between race, household income and grip strength in middle- and older-aged adults. Ethn. Dis..

[17] Yun I, Park YS, Park E-C, Jang S-I (2022). Association between changes in working status and hand-grip strength among Korean middle-aged and older adults: A longitudinal panel study. Sci. Rep..

[18] Firth J, Stubbs B, Vancampfort D, Firth JA, Large M, Rosenbaum S (2018). Grip strength is associated with cognitive performance in schizophrenia and the general population: A UK biobank study of 476559 participants. Schizophr. Bull..

[19] Jeong S, Choi S, Kim K, Kim SM, Kim S, Park SM (2018). Association among handgrip strength, body mass index and decline in cognitive function among the elderly women. BMC Geriatr..

[20] Massy-Westropp NM, Gill TK, Taylor AW, Bohannon RW, Hill CL (2011). Hand Grip Strength: Age and gender stratified normative data in a population-based study. BMC Res. Notes..

[21] Sternäng O, Reynolds CA, Finkel D, Ernsth-Bravell M, Pedersen NL, Dahl Aslan AK (2016). Grip strength and cognitive abilities: Associations in old age. J. Gerontol. B Psychol. Sci. Soc. Sci..

[22] Mendes J, Amaral TF, Borges N, Santos A, Padrão P, Moreira P (2017). Handgrip strength values of Portuguese older adults: A population based study. BMC Geriatr..

[23] Ong HL, Abdin E, Chua BY, Zhang Y, Seow E, Vaingankar JA (2017). Hand-grip strength among older adults in Singapore: A comparison with international norms and associative factors. BMC Geriatr..

[24] Ramlagan S, Peltzer K, Phaswana-Mafuya N (2014). Hand grip strength and associated factors in non-institutionalised men and women 50 years and older in South Africa. BMC Res. Notes..

[25] Forrest KYZ, Williams AM, Leeds MJ, Robare JF, Bechard TJ (2018). Patterns and correlates of grip strength in older Americans. Curr. Aging Sci..

[26] Ahn H, Choi HY, Ki M (2022). Association between levels of physical activity and low handgrip strength: Korea National Health and Nutrition Examination Survey 2014–2019. Epidemiol. Health..

[27] Lee S-Y, Son D-H, Lee Y-J (2020). Relationship between sedentary time and handgrip strength in healthy korean women: Korea National Health and Nutrition Examination Survey 2014–2016. Korean J. Fam. Med..

[28] Woo J, Goggins W, Sham A, Ho SC (2005). Social determinants of frailty. Gerontology..

[29] Arokiasamy P, Selvamani Y (2018). Age, socioeconomic patterns and regional variations in grip strength among older adults (50+) in India: Evidence from WHO’s Study on Global Ageing and Adult Health (SAGE). Arch. Gerontol. Geriatr..

[30] Hoogendijk EO, van Hout HPJ, Heymans MW, van der Horst HE, Frijters DHM, Broese van Groenou MI (2014). Explaining the association between educational level and frailty in older adults: Results from a 13-year longitudinal study in the Netherlands. Ann. Epidemiol..

[31] Ukegbu U, Maselko J, Malhotra R, Perera B, Østbye T (2014). Correlates of hand grip strength and activities of daily living in Elderly Sri Lankans. J. Am. Geriatr. Soc..

[32] Yorke AM, Curtis AB, Shoemaker M, Vangsnes E (2017). The impact of multimorbidity on grip strength in adults age 50 and older: Data from the health and retirement survey (HRS). Arch. Gerontol. Geriatr..

[33] Selvamani Y, Arokiasamy P, Chaudhary M (2018). Association of sleep problems and sleep duration with self-rated health and grip strength among older adults in India and China: Results from the study on global aging and adult health (SAGE). J. Public Health (Berl.)..

[34] Smith L, White S, Stubbs B, Hu L, Veronese N, Vancampfort D (2018). Depressive symptoms, handgrip strength, and weight status in US older adults. J. Affect. Disord..

[35] Etman A, Kamphuis CBM, van der Cammen TJM, Burdorf A, van Lenthe FJ (2015). Do lifestyle, health and social participation mediate educational inequalities in frailty worsening?. Eur. J. Public Health..

[36] Mezuk B, Edwards L, Lohman M, Choi M, Lapane K (2012). Depression and frailty in later life: A synthetic review. Int. J. Geriatr. Psychiatry..

[37] Tyrovolas S, Koyanagi A, Olaya B, Ayuso-Mateos JL, Miret M, Chatterji S (2016). Factors associated with skeletal muscle mass, sarcopenia, and sarcopenic obesity in older adults: A multi-continent study. J. Cachexia Sarcopenia Muscle..

[38] International Institute for Population Sciences (IIPS), NPHCE, MoHFW, Harvard T. H. Chan School of Public Health (HSPH), The University of Southern California (USC). Longitudinal Ageing Study in India (LASI) Wave 1. Mumbai, India (2020).

[39] Gunasekaran V, Banerjee J, Dwivedi SN, Upadhyay AD, Chatterjee P, Dey AB (2016). Normal gait speed, grip strength and thirty seconds chair stand test among older Indians. Arch. Gerontol. Geriatr..

[40] Muhammad T, Hossain B, Das A, Rashid M (2022). Relationship between handgrip strength and self-reported functional difficulties among older Indian adults: The role of self-rated health. Exp. Gerontol..

[41] Muhammad T, Maurya P (2022). Relationship between handgrip strength, depression and cognitive functioning among older adults: Evidence from longitudinal ageing study in India. Int. J. Geriatr. Psychiatry.

[42] Chen L-K, Woo J, Assantachai P, Auyeung T-W, Chou M-Y, Iijima K (2020). Asian Working Group for Sarcopenia: 2019 consensus update on sarcopenia diagnosis and treatment. J. Am. Med. Directors Assoc..

[43] Hu P, Wang S, Lee J (2017). Socioeconomic gradients of cardiovascular risk factors in China and India: Results from the China health and retirement longitudinal study and longitudinal aging study in India. Int. J. Public Health..

[44] Rutstein, S.O., Staveteig, S. Making the demographic and health surveys wealth index comparable. (2014).

[45] Vidal, R., Ma, Y., Sastry, S.S. Principal component analysis. in *Interdisciplinary Applied Mathematics*. (2016).

[46] Muhammad T, Skariah AE, Kumar M, Srivastava S (2022). Socioeconomic and health-related inequalities in major depressive symptoms among older adults: A Wagstaff’s decomposition analysis of data from the LASI baseline survey, 2017–2018. BMJ open..

[47] Adler NE, Epel ES, Castellazzo G, Ickovics JR (2000). Relationship of subjective and objective social status with psychological and physiological functioning: Preliminary data in healthy, White women. Health Psychol..

[48] Hooker ED, Campos B, Hoffman L, Zoccola P, Dickerson SS (2020). Is receiving social support costly for those higher in subjective socioeconomic status?. Int. J. Behav. Med..

[49] Suchday S, Chhabra R, Wylie-Rosett J, Almeida M (2008). Subjective and objective measures of socioeconomic status: Predictors of cardiovascular risk in college students in Mumbai, India. Ethnicity Disease..

[50] Muhammad T, Sekher TV, Srivastava S (2022). Association of objective and subjective socioeconomic markers with cognitive impairment among older adults: Cross-sectional evidence from a developing country. BMJ Open..

[51] Srivastava S, Sulaiman KM, Drishti D, Muhammad T (2021). Factors associated with psychiatric disorders and treatment seeking behaviour among older adults in India. Sci. Rep..

[52] Muhammad T, Sulaiman MK, Srivastava S (2022). Migration of adult male children and associated depression among community-dwelling older parents: A cross-sectional gender analysis from Longitudinal Ageing Study in India, 2017–2018. Int. J. Geriatr. Psychiatry.

[53] Nayar KR (2007). Social exclusion, caste & health: A review based on the social determinants framework. Indian J. Med. Res..

[54] Srivastava S, Purkayastha N, Chaurasia H, Muhammad T (2021). Socioeconomic inequality in psychological distress among older adults in India: A decomposition analysis. BMC Psychiatry..

[55] Kakwani N, Wagstaff A, Van Doorslaer E (1997). Socioeconomic inequalities in health: Measurement, computation, and statistical inference. J. Econ..

[56] Konings P, Harper S, Lynch J, Hosseinpoor AR, Berkvens D, Lorant V (2010). Analysis of socioeconomic health inequalities using the concentration index. Int. J. Public Health..

[57] Wagstaff A (2000). Socioeconomic inequalities in child mortality: Comparisons across nine developing countries. Bull. World Health Organ..

[58] Borah G (2021). Gender gap in life expectancy in India and role of age groups: A comparison between before and after male–female life expectancy at birth crossover. PLOS ONE..

[59] Frederiksen H, Hjelmborg J, Mortensen J, McGue M, Vaupel JW, Christensen K (2006). Age trajectories of grip strength: Cross-sectional and longitudinal data among 8,342 Danes aged 46 to 102. Ann. Epidemiol..

[60] Oksuzyan A, Maier H, McGue M, Vaupel JW, Christensen K (2010). Sex differences in the level and rate of change of physical function and grip strength in the Danish 1905-cohort study. J. Aging Health..

[61] Pan P-J, Lin C-H, Yang N-P, Chen H-C, Tsao H-M, Chou P (2020). Normative data and associated factors of hand grip strength among elderly individuals: The Yilan Study, Taiwan. Sci. Rep..

[62] Stenholm S, Tiainen K, Rantanen T, Sainio P, Heliövaara M, Impivaara O (2012). Long-term determinants of muscle strength decline: Prospective evidence from the 22-year mini-Finland follow-up survey. J. Am. Geriatr. Soc..

[63] Sternäng O, Reynolds CA, Finkel D, Ernsth-Bravell M, Pedersen NL, Dahl Aslan AK (2015). Factors associated with grip strength decline in older adults. Age Ageing..

[64] Alley DE, Shardell MD, Peters KW, McLean RR, Dam T-TL, Kenny AM (2014). Grip strength cutpoints for the identification of clinically relevant weakness. J. Gerontol. Series A Biomed. Sci. Med. Sci..

[65] Muollo V, Tatangelo T, Ghiotto L, Cavedon V, Milanese C, Zamboni M (2022). Is handgrip strength a marker of muscle and physical function of the lower limbs? Sex differences in older adults with obesity. Nutr. Metab. Cardiovasc. Diseases..

[66] Shidhaye R, Patel V (2010). Association of socio-economic, gender and health factors with common mental disorders in women: A population-based study of 5703 married rural women in India. Int. J. Epidemiol..

[67] Lund C, Breen A, Flisher AJ, Kakuma R, Corrigall J, Joska JA, Swartz L, Patel V (2010). Poverty and common mental disorders in low and middle income countries: A systematic review. Soc. Sci. Med..

[68] Patel V, Kirkwood BR, Pednekar S, Pereira B, Barros P, Fernandes J (2006). Gender disadvantage and reproductive health risk factors for common mental disorders in women: A community survey in India. Arch. General Psychiatry..

[69] Connell, R.W. The Men and the Boys. (2001).

[70] Dolan A (2011). “You can’t ask for a Dubonnet and lemonade!”: Working class masculinity and men’s health practices. Sociol Health Illn..

[71] Griffith, D. M., Gilbert, K. L., Bruce, M. A. & Thorpe, R. J. Masculinity in men’s health: Barrier or portal to healthcare?. in *Men’s Health in Primary Care* 19–31 (2016).

[72] Shozi S, Monyeki MA, Moss SJ, Pienaar C (2022). Relationships between physical activity, body mass index, waist circumference and handgrip strength amongst adults from the North West province, South Africa: The PURE study. Afr. J. Prim. Health Care Fam. Med..

[73] Leino-Arjas P, Solovieva S, Riihimäki H, Kirjonen J, Telama R (2004). Leisure time physical activity and strenuousness of work as predictors of physical functioning: A 28 year follow up of a cohort of industrial employees. Occup. Environ. Med..

[74] Richardson N (2010). ‘The ‘Buck’ Stops with Me’-reconciling men’s lay conceptualisations of responsibility for health with men's health policy. Health Sociol. Rev. J. Health Sect. Aust. Sociol. Assoc..

[75] Barbat-Artigas S, Rolland Y, Vellas B, Aubertin-Leheudre M (2013). Muscle quantity is not synonymous with muscle quality. J. Am. Med. Dir. Assoc..

[76] Selvamani Y, Singh P (2018). Socioeconomic patterns of underweight and its association with self-rated health, cognition and quality of life among older adults in India. PLoS One..

[77] Saito T, Miyatake N, Sakano N, Oda K, Katayama A, Nishii K (2012). Relationship between cigarette smoking and muscle strength in Japanese men. J. Prev. Med. Public Health..

[78] Kojima G, Iliffe S, Walters K (2015). Smoking as a predictor of frailty: A systematic review. BMC Geriatr..

[79] Kim HI, Lee HS, Lee SW, Shim KW, Ryou I, Jeong YH (2020). Hand grip strength according to the smoking status in Korean adults: The 7th Korea National Health and Nutrition Examination Survey 2016–2017. Korean J. Fam. Practice..

[80] Hubbard RE, Searle SD, Mitnitski A, Rockwood K (2009). Effect of smoking on the accumulation of deficits, frailty and survival in older adults: A secondary analysis from the Canadian Study of Health and Aging. J. Nutr. Health Aging..

[81] Degens H, Gayan-Ramirez G, van Hees HWH (2015). Smoking-induced skeletal muscle dysfunction: From evidence to mechanisms. Am. J. Respir. Crit. Care Med..

[82] Petersen AMW, Magkos F, Atherton P, Selby A, Smith K, Rennie MJ (2007). Smoking impairs muscle protein synthesis and increases the expression of myostatin and MAFbx in muscle. Am. J. Physiol. Endocrinol. Metab..

[83] Demakakos P, Nazroo J, Breeze E, Marmot M (2008). Socioeconomic status and health: The role of subjective social status. Soc. Sci. Med..

[84] Singh-Manoux A, Marmot MG, Adler NE (2005). Does subjective social status predict health and change in health status better than objective status?. Psychosom. Med..

[85] Präg P (2020). Subjective socio-economic status predicts self-rated health irrespective of objective family socio-economic background. Scand. J. Public Health..

[86] English AN, Bellingtier JA, Neupert SD (2019). It’s “the Joneses”: The influence of objective and subjective socioeconomic status on subjective perceptions of aging. Eur. J. Ageing..

[87] Euteneuer F (2014). Subjective social status and health. Curr. Opin. Psychiatry..

[88] Huang S, Hou J, Sun L, Dou D, Liu X, Zhang H (2017). The effects of objective and subjective socioeconomic status on subjective well-being among rural-to-urban migrants in China: The moderating role of subjective social mobility. Front. Psychol..

[89] Gong F, Xu J, Takeuchi DT (2012). Beyond conventional socioeconomic status: Examining subjective and objective social status with self-reported health among Asian immigrants. J. Behav. Med..

[90] Frerichs L, Huang TT-K, Chen D-R (2014). Associations of subjective social status with physical activity and body mass index across Four Asian Countries. J. Obes..

[91] Adler NE, Epel ES, Castellazzo G, Ickovics JR (2000). Relationship of subjective and objective social status with psychological and physiological functioning: Preliminary data in healthy white women. Health Psychol..

[92] Yue, Y., Jia, Y., Wang, X. Grip strength asymmetry and activities of daily living in middle-aged and elderly Chinese Relevance studies. Review. (2022).

[93] Raji MA, Kuo Y-F, Snih SA, Markides KS, Peek MK, Ottenbacher KJ (2005). Cognitive status, muscle strength, and subsequent disability in older Mexican Americans. J. Am. Geriatr. Soc..

[94] Wang J, Zhou X, Qiu S, Deng L, Li J, Yang L (2005). The association between grip strength and depression among adults aged 60 years and older: A large-scaled population-based study from the longitudinal aging study in India. Front. Aging Neurosci..

[95] White HK, McConnell ES, Bales CW, Kuchibhatla M (2004). A 6-month observational study of the relationship between weight loss and behavioral symptoms in institutionalized Alzheimer’s disease subjects. J. Am. Med. Dir. Assoc..

[96] Visser M, Pahor M, Taaffe DR, Goodpaster BH, Simonsick EM, Newman AB (2002). Relationship of interleukin-6 and tumor necrosis factor-with muscle mass and muscle strength in elderly men and women: The health ABC Study. J. Gerontol. Series A Biol. Sci. Med. Sci..

[97] Weaver JD, Huang M-H, Albert M, Harris T, Rowe JW, Seeman TE (2002). Interleukin-6 and risk of cognitive decline: MacArthur studies of successful aging. Neurology..

[98] Alqahtani B, Alenazi A, Alshehri M, Alqahtani M, Elnaggar R (2019). Reference values and associated factors of hand grip strength in elderly Saudi population: A cross-sectional study. BMC Geriatr..

[99] Carney C, Benzeval M (2018). Social patterning in grip strength and in its association with age; a cross sectional analysis using the UK Household Longitudinal Study (UKHLS). BMC Public Health..

[100] Pothisiri W, Prasitsiriphon O, Saikia N, Aekplakorn W (2021). Education and grip strength among older Thai adults: A mediation analysis on health-related behaviours. SSM Popul. Health..

[101] Satheannoppakao W, Aekplakorn W, Pradipasen M (2009). Fruit and vegetable consumption and its recommended intake associated with sociodemographic factors: Thailand National Health Examination Survey III. Public Health Nutr..

[102] Kaplan MS, Newsom JT, McFarland BH, Lu L (2001). Demographic and psychosocial correlates of physical activity in late life. Am. J. Prevent. Med..

[103] Shaw BA, Spokane LS (2008). Examining the association between education level and physical activity changes during early old age. J. Aging Health..

[104] Cutler DM, Lleras-Muney A (2010). Understanding differences in health behaviors by education. J. Health Econ..

[105] Margerison-Zilko C, Cubbin C (2013). Socioeconomic disparities in tobacco-related health outcomes across racial/ethnic groups in the United States: National Health Interview Survey 2010. Nicotine Tob. Res..

[106] George LK (2005). Socioeconomic status and health across the life course: Progress and prospects. J. Gerontol. Series B..

[107] Gross AL, Parisi JM, Spira AP, Kueider AM, Ko JY, Saczynski JS (2012). Memory training interventions for older adults: A meta-analysis. Aging Ment. Health..

