# Supplementary material for: Examining factors contributing to the socioeconomic inequalities in handgrip strength among older adults in India: a decomposition analysis
Source: Sci Rep. 2024 Jan 3;14:442. doi: 10.1038/s41598-023-50613-3 (PMC10764745; doi:10.1038/s41598-023-50613-3)
Supplement: Supplementary file 1 — Supplementary Tables. [file 41598_2023_50613_MOESM1_ESM.docx]

| Table S1: Multivariable logistic regression estimates of weak grip strength by background variables among older adults, stratified by sex, LASI-2017-18 | | | |
| --- | --- | --- | --- |
| Variable | Categories | Male (n=11,637) | Female (n=11,374) |
|  |  | Adjusted odds ratio (95% CI) | Adjusted odds ratio (95% CI) |
| Age | 60-69 years | Ref. | Ref. |
|  | 70-79 years | 0.71*** (0.58 - 0.86) | 0.80 (0.53 - 1.22) |
|  | 80 years & above | 1.64* (1.12 - 2.39) | 1.96** (1.25 - 3.09) |
| Marital status | Married | Ref. | Ref. |
|  | Widowed | 1.09 (0.86 - 1.37) | 1.38 (0.89 - 2.13) |
|  | Divorced and others | 0.84 (0.49 - 1.44) | 0.96 (0.38 - 2.45) |
| Education | No | Ref. | Ref. |
|  | Primary | 0.78* (0.61 - 0.98) | 1.18 (0.71 - 1.96) |
|  | Secondary | 0.74* (0.58 - 0.95) | 0.80 (0.40 - 1.60) |
|  | Higher | 0.94 (0.63 - 1.41) | 1.27 (0.39 - 4.20) |
| Work status | Never worked | Ref. | Ref. |
|  | Not working | 1.09 (0.73 - 1.65) | 1.72** (1.14 - 2.57) |
|  | Working | 0.74 (0.49 - 1.13) | 0.74 (0.39 - 1.42) |
|  | Retired | 1.07 (0.68 - 1.70) | 0.91 (0.28 - 2.96) |
| Alcohol | No | Ref. | Ref. |
|  | Yes | 1.08 (0.81 - 1.45) | 0.96 (0.37 - 2.44) |
| Smoking | No | Ref. | Ref. |
|  | Yes | 0.70** (0.57 - 0.87) | 2.15* (1.19 - 3.89) |
| Chewing tobacco | No | Ref. | Ref. |
|  | Yes | 0.87 (0.70 - 1.08) | 0.67 (0.40 - 1.14) |
| Vigorous activity | No | Ref. | Ref. |
|  | Yes | 0.81* (0.66 - 0.99) | 1.30 (0.81 - 2.09) |
| Moderate activity | No | Ref. | Ref. |
|  | Yes | 0.82 (0.56 - 1.20) | 0.59 (0.19 - 1.83) |
| Yoga-related activity | No | Ref. | Ref. |
|  | Yes | 0.66** (0.50 - 0.88) | 0.65 (0.30 - 1.42) |
| Multi-morbidity | No | Ref. | Ref. |
|  | Yes | 0.86 (0.67 - 1.10) | 1.15 (0.75 - 1.77) |
| ADL difficulty | No | Ref. | Ref. |
|  | Yes | 1.54*** (1.21 - 1.96) | 1.25 (0.82 - 1.90) |
| IADL difficulty | No | Ref. | Ref. |
|  | Yes | 1.39*** (1.14 - 1.68) | 1.60* (1.03 - 2.49) |
| Poor SRH | No | Ref. | Ref. |
|  | Yes | 1.30* (1.04 - 1.61) | 1.05 (0.67 - 1.65) |
| Depression | No | Ref. | Ref. |
|  | Yes | 0.89 (0.64 - 1.23) | 0.58 (0.33 - 1.02) |
| Cognitive dysfunction | No | Ref. | Ref. |
|  | Yes | 1.21 (0.89 - 1.63) | 1.63* (1.09 - 2.45) |
| BMI | Normal | Ref. | Ref. |
|  | Underweight | 1.64*** (1.35 - 1.99) | 1.65* (1.09 - 2.52) |
|  | Overweight | 0.77 (0.56 - 1.06) | 0.51* (0.30 - 0.86) |
|  | Obese | 0.68 (0.38 - 1.21) | 1.05 (0.54 - 2.02) |
| Wealth quintiles (MPCE) | Poorest | Ref. | Ref. |
|  | Poor | 1.03 (0.80 - 1.33) | 0.87 (0.49 - 1.52) |
|  | Middle | 1.10 (0.83 - 1.46) | 1.13 (0.66 - 1.94) |
|  | Rich | 0.99 (0.75 - 1.30) | 1.34 (0.77 - 2.36) |
|  | Richest | 0.99 (0.74 - 1.33) | 1.34 (0.75 - 2.41) |
| Subjective social status | Low | Ref. | Ref. |
|  | High | 0.80* (0.67 - 0.95) | 0.89 (0.63 - 1.25) |
| Social group | SC | Ref. | Ref. |
|  | ST | 0.79 (0.57 - 1.11) | 1.21 (0.54 - 2.73) |
|  | OBC | 0.88 (0.71 - 1.09) | 0.81 (0.51 - 1.30) |
|  | Others | 0.75* (0.56 - 0.99) | 1.70* (1.05 - 2.75) |
| Place of residence | Urban | Ref. | Ref. |
|  | Rural | 0.97 (0.78 - 1.20) | 1.03 (0.66 - 1.59) |
| Religion | Hindu | Ref. | Ref. |
|  | Muslim | 0.76 (0.57 - 1.01) | 0.72 (0.37 - 1.40) |
|  | Christian | 0.97 (0.60 - 1.55) | 1.02 (0.54 - 1.93) |
|  | Others | 0.73 (0.45 - 1.18) | 1.73 (0.70 - 4.24) |
| Region | North | Ref. | Ref. |
|  | Central | 0.62** (0.45 - 0.84) | 1.33 (0.63 - 2.82) |
|  | East | 1.25 (0.93 - 1.69) | 1.60 (0.76 - 3.36) |
|  | North-east | 0.76 (0.52 - 1.13) | 0.72 (0.29 - 1.76) |
|  | West | 2.77*** (2.09 - 3.67) | 5.19*** (2.76 - 9.75) |
|  | South | 1.76*** (1.26 - 2.46) | 3.07** (1.53 - 6.15) |

ADL: Activities of daily living; IADL: Instrumental activities of daily living; SRH: Self-rated health; BMI: Body mass index; MPCE: Monthly per capita consumption expenditure

| Table S2: Sensitivity analysis of multivariable logistic regression estimates of weak grip strength by background variables among older adults, after excluding those in the age group 80+ years (n=3,389), LASI-2017-18 | | |
| --- | --- | --- |
| Variable | Categories | Adjusted odds ratio (95% CI) |
| Age | 60-69 years | Ref. |
|  | 70-79 years | 0.71*** (0.60 - 0.84) |
| Sex | Male | Ref. |
|  | Female | 0.083*** (0.063 - 0.11) |
| Marital status | Married | Ref. |
|  | Widowed | 1.20 (0.97 - 1.48) |
|  | Divorced and others | 1.02 (0.63 - 1.65) |
| Education | No | Ref. |
|  | Primary | 0.80 (0.64 - 1.00) |
|  | Secondary | 0.75* (0.59 - 0.95) |
|  | Higher | 0.80 (0.55 - 1.15) |
| Work status | Never worked | Ref. |
|  | Not working | 1.27 (0.92 - 1.75) |
|  | Working | 0.83 (0.60 - 1.15) |
|  | Retired | 1.10 (0.73 - 1.64) |
| Alcohol | No | Ref. |
|  | Yes | 1.12 (0.85 - 1.48) |
| Smoking | No | Ref. |
|  | Yes | 0.75** (0.61 - 0.93) |
| Chewing tobacco | No | Ref. |
|  | Yes | 0.92 (0.76 - 1.13) |
| Vigorous activity | No | Ref. |
|  | Yes | 0.78** (0.66 - 0.92) |
| Moderate activity | No | Ref. |
|  | Yes | 0.71* (0.52 - 0.98) |
| Yoga-related activity | No | Ref. |
|  | Yes | 0.78* (0.61 - 1.00) |
| Multi-morbidity | No | Ref. |
|  | Yes | 1.02 (0.82 - 1.26) |
| ADL difficulty | No | Ref. |
|  | Yes | 1.58*** (1.28 - 1.94) |
| IADL difficulty | No | Ref. |
|  | Yes | 1.36*** (1.14 - 1.62) |
| Poor SRH | No | Ref. |
|  | Yes | 1.23* (1.02 - 1.50) |
| Depression | No | Ref. |
|  | Yes | 0.90 (0.68 - 1.20) |
| Cognitive dysfunction | No | Ref. |
|  | Yes | 1.25 (0.95 - 1.63) |
| BMI | Normal | Ref. |
|  | Underweight | 1.63*** (1.36 - 1.96) |
|  | Overweight | 0.67** (0.51 - 0.87) |
|  | Obese | 1.00 (0.67 - 1.49) |
| Wealth quintiles (MPCE) | Poorest | Ref. |
|  | Poor | 0.90 (0.71 - 1.15) |
|  | Middle | 0.93 (0.73 - 1.20) |
|  | Rich | 0.98 (0.76 - 1.26) |
|  | Richest | 0.94 (0.71 - 1.24) |
| Subjective social status | Low | Ref. |
|  | High | 0.78** (0.66 - 0.93) |
| Social group | SC | Ref. |
|  | ST | 0.86 (0.61 - 1.19) |
|  | OBC | 0.86 (0.70 - 1.05) |
|  | Others | 0.85 (0.65 - 1.11) |
| Place of residence | Urban | Ref. |
|  | Rural | 1.04 (0.85 - 1.27) |
| Religion | Hindu | Ref. |
|  | Muslim | 0.75* (0.56 - 0.99) |
|  | Christian | 0.88 (0.57 - 1.36) |
|  | Others | 0.73 (0.45 - 1.19) |
| Region | North | Ref. |
|  | Central | 0.69* (0.50 - 0.94) |
|  | East | 1.28 (0.97 - 1.71) |
|  | North-east | 0.76 (0.52 - 1.13) |
|  | West | 2.80*** (2.15 - 3.66) |
|  | South | 1.84*** (1.35 - 2.52) |

ADL: Activities of daily living; IADL: Instrumental activities of daily living; SRH: Self-rated health; BMI: Body mass index; MPCE: Monthly per capita consumption expenditure

| Table S3: Sensitivity analysis of multivariable logistic regression estimates of weak grip strength (Asian cut-point) by background variables among older adults, LASI-2017-18 | | | |
| --- | --- | --- | --- |
| Variable | Categories | Crude odds ratio (95% CI) | Adjusted odds ratio (95% CI) |
| Age | 60-69 years | Ref. | Ref. |
|  | 70-79 years | 2.88*** (2.55 - 3.24) | 2.41*** (2.09 - 2.79) |
|  | 80 years & above | 6.61*** (4.97 - 8.81) | 4.12*** (2.89 - 5.87) |
| Sex | Male | Ref. | Ref. |
|  | Female | 0.81*** (0.73 - 0.90) | 0.58*** (0.50 - 0.67) |
| Marital status | Married | Ref. | Ref. |
|  | Widowed | 1.45*** (1.26 - 1.67) | 1.00 (0.86 - 1.17) |
|  | Divorced and others | 1.28* (0.98 - 1.65) | 0.88 (0.66 - 1.18) |
| Education | No | Ref. | Ref. |
|  | Primary | 0.89* (0.79 - 1.00) | 0.91 (0.79 - 1.05) |
|  | Secondary | 0.67*** (0.55 - 0.81) | 0.78** (0.62 - 0.97) |
|  | Higher | 0.53*** (0.45 - 0.63) | 0.66*** (0.51 - 0.84) |
| Work status | Never worked | Ref. | Ref. |
|  | Not working | 1.85*** (1.57 - 2.18) | 1.28*** (1.07 - 1.52) |
|  | Working | 0.90 (0.77 - 1.06) | 0.87 (0.72 - 1.05) |
|  | Retired | 0.97 (0.80 - 1.19) | 0.98 (0.77 - 1.24) |
| Alcohol | No | Ref. | Ref. |
|  | Yes | 1.26*** (1.07 - 1.49) | 1.07 (0.88 - 1.30) |
| Smoking | No | Ref. | Ref. |
|  | Yes | 1.07 (0.95 - 1.22) | 0.78*** (0.66 - 0.92) |
| Chewing tobacco | No | Ref. | Ref. |
|  | Yes | 1.02 (0.91 - 1.13) | 0.84*** (0.74 - 0.95) |
| Vigorous activity | No | Ref. | Ref. |
|  | Yes | 0.66*** (0.60 - 0.74) | 0.85*** (0.75 - 0.96) |
| Moderate activity | No | Ref. | Ref. |
|  | Yes | 0.76*** (0.65 - 0.89) | 0.98 (0.83 - 1.16) |
| Yoga-related activity | No | Ref. | Ref. |
|  | Yes | 0.66*** (0.59 - 0.74) | 0.98 (0.85 - 1.13) |
| Multi-morbidity | No | Ref. | Ref. |
|  | Yes | 0.99 (0.84 - 1.18) | 0.94 (0.80 - 1.10) |
| ADL difficulty | No | Ref. | Ref. |
|  | Yes | 1.83*** (1.60 - 2.10) | 1.21** (1.03 - 1.41) |
| IADL difficulty | No | Ref. | Ref. |
|  | Yes | 1.75*** (1.55 - 1.97) | 1.22*** (1.05 - 1.41) |
| Poor SRH | No | Ref. | Ref. |
|  | Yes | 1.70*** (1.52 - 1.89) | 1.21** (1.04 - 1.41) |
| Depression | No | Ref. | Ref. |
|  | Yes | 1.11 (0.95 - 1.29) | 1.00 (0.84 - 1.18) |
| Cognitive dysfunction | No | Ref. | Ref. |
|  | Yes | 2.12*** (1.82 - 2.47) | 1.48*** (1.25 - 1.75) |
| BMI | Normal | Ref. | Ref. |
|  | Underweight | 2.07*** (1.85 - 2.32) | 2.07*** (1.81 - 2.36) |
|  | Overweight | 0.66*** (0.55 - 0.79) | 0.69*** (0.59 - 0.81) |
|  | Obese | 0.65** (0.45 - 0.93) | 0.71** (0.51 - 1.00) |
| Wealth quintiles (MPCE) | Poorest | Ref. | Ref. |
|  | Poor | 0.91 (0.80 - 1.05) | 1.07 (0.92 - 1.25) |
|  | Middle | 0.84** (0.73 - 0.96) | 1.00 (0.85 - 1.17) |
|  | Rich | 0.80** (0.65 - 0.97) | 1.02 (0.83 - 1.26) |
|  | Richest | 0.75*** (0.64 - 0.87) | 1.07 (0.90 - 1.27) |
| Subjective social status | Low | Ref. | Ref. |
|  | High | 0.82*** (0.73 - 0.91) | 0.97 (0.84 - 1.13) |
| Social group | SC | Ref. | Ref. |
|  | ST | 1.00 (0.84 - 1.19) | 0.91 (0.72 - 1.16) |
|  | OBC | 0.94 (0.81 - 1.09) | 0.87* (0.74 - 1.03) |
|  | Others | 0.71*** (0.62 - 0.81) | 0.84** (0.71 - 1.00) |
| Place of residence | Urban | Ref. | Ref. |
|  | Rural | 0.77*** (0.64 - 0.92) | 0.84*** (0.73 - 0.96) |
| Religion | Hindu | Ref. | Ref. |
|  | Muslim | 1.10 (0.94 - 1.28) | 0.95 (0.81 - 1.12) |
|  | Christian | 0.92 (0.81 - 1.05) | 1.40** (1.08 - 1.82) |
|  | Others | 1.55*** (1.27 - 1.88) | 0.88 (0.71 - 1.09) |
| Region | North | Ref. | Ref. |
|  | Central | 1.22*** (1.09 - 1.38) | 1.06 (0.91 - 1.24) |
|  | East | 1.35*** (1.20 - 1.52) | 1.27*** (1.08 - 1.48) |
|  | North-east | 0.89* (0.78 - 1.02) | 0.82** (0.68 - 0.98) |
|  | West | 2.20*** (1.72 - 2.80) | 2.36*** (1.92 - 2.89) |
|  | South | 1.58*** (1.38 - 1.82) | 1.83*** (1.54 - 2.18) |

ADL: Activities of daily living; IADL: Instrumental activities of daily living; SRH: Self-rated health; BMI: Body mass index; MPCE: Monthly per capita consumption expenditure
